# Dissecting Causal Relationships Between Gut Microbiota, Immunocyte Phenotype, and Migraine: A Mendelian Randomization Study

**DOI:** 10.1002/brb3.70693

**Published:** 2025-07-11

**Authors:** Yupei Lai, Yike Liu, Jiahao Chen, Yu Cao, Xiangsheng Zhang, Lu Li, Pengyu Zhou, Peng Sun, Jun Zhou

**Affiliations:** ^1^ The Third Affiliated Hospital of Southern Medical University Guangzhou China; ^2^ Shunde Hospital of Guangzhou University of Chinese Medicine Foshan China; ^3^ Deparment of Anesthesiology Sun Yat‐sen University Cancer Center Guangzhou China

**Keywords:** gut microbiota, immune traits, Mendelian Randomization, migraine

## Abstract

**Background:**

Migraines impose a substantial economic and societal burden, yet their underlying mechanisms remain poorly understood. While observational studies suggest associations between gut microbiota dysbiosis, immunophenotypic alterations, and migraine risk, causal evidence is lacking. This study leverages Mendelian Randomization to investigate the causal relationship between gut microbiota and migraine while exploring the mediating role of immune traits in this association.

**Methods:**

We employed a two‐sample, two‐step Mendelian Randomization approach to examine the mediating effects of immunocyte phenotypes on the relationship between gut microbiota and migraine outcomes, including migraine with aura and migraine without aura. The primary analysis utilized inverse‐variance weighted estimation, supplemented by sensitivity analyses to ensure robustness. Summary statistics for gut microbiota were sourced from the MiBioGen consortium and NHGRI‐EBI, immunocyte phenotypes from the GWAS catalog, and migraine data from the FinnGen consortium. Next, we evaluated *Prevotella histicola* level by qPCR.

**Results:**

Our analysis identified suggestive associations between 34 gut microbiota taxa and migraine subtypes. Notably, Family *Bifidobacteriaceae* (id.433, PIVW: 1.51×10⁻⁵, ORIVW: 0.861, 95% CI: 0.765–0.970) and Order *Bifidobacteriales* (id.432, PIVW: 1.51×10⁻⁵, ORIVW: 0.861, 95% CI: 0.765–0.970) demonstrated strong causal links to a reduced risk of migraine. Mediation analysis revealed that HLA‐DR on monocytes mediated 7.74% of the causal pathway from Genus *Prevotella9* (id.11183, PIVW: 0.002, ORIVW: 0.834, 95% CI: 0.744–0.936) to MA.

**Conclusions:**

This study provides robust evidence of a causal relationship between *Bifidobacteriaceae* and a decreased risk of migraine. Furthermore, we identify HLA‐DR on monocytes as a key inflammatory mediator in the protective effect of *Prevotella* against migraine with aura. These findings highlight the potential of gut microbiota modulation and immune‐targeted therapies in migraine prevention and treatment, offering novel insights into the gut‐brain‐immune axis in migraine pathogenesis.

## Graphical abstract

This MR analysis identified suggestive associations between 34 gut microbiota taxa and migraine subtypes while exploring the mediating role of immune traits in this association. Experimental results demonstrate that the abundance of *Prevotella histicola* is reduced in migraine model mice.

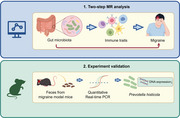

## 1 Introduction

Migraine is a neurological disorder characterized by recurring one‐sided headaches and symptoms like vertigo, dizziness, tinnitus, and cognitive issues (Raggi et al. 2024). It significantly impacts patients' well‐being and poses an economic burden on families and society (Steiner et al. 2024; Peres et al. 2024). Despite its prevalence, the cause of migraines is unclear, and treatment options are limited, making it crucial to understand its risk factors and mechanisms for better prevention and treatment.

Recent studies highlight the significant role of the gut‐brain axis in neurological disorders such as schizophrenia, Parkinson's, and epilepsy (Socała et al. 2021; Zhou et al. 2024). At present, observational studies link migraines to gut flora imbalances, with a higher incidence of gastrointestinal issues like inflammatory bowel disease, celiac disease, and irritable bowel syndrome in migraine sufferers (van Hemert et al. 2014). Animal research shows that germ‐free mice, which lack native microbiota, exhibit reduced pain from inflammation but develop migraine‐like pain after nitroglycerin treatment. Fecal microbiota transplantation can alleviate this pain, indicating that gut microbiota imbalance is crucial in migraine development (Amaral et al. 2008; Tang et al. 2020). Currently, although some researchers have explored the causal link between migraines and gut microbiota using Mendelian randomization, the studies are limited by small sample sizes and a single analysis method. Consequently, further elucidation of the precise causal relationship between gut microbiota and migraine, along with the identification of mediating factors, could offer novel insights into diagnosis and treatment strategies.

Disruption of gut microbiota can impact the immune system and contribute to systemic diseases. Research indicates a strong correlation between intestinal microbiota and migraine, suggesting that alterations in immune characteristics may play a crucial role in this association (Zhou et al. 2024). However, it is still controversial that inflammatory factors mediate the occurrence of migraine. A study found that migraine patients had fewer regulatory T cells in their blood compared to healthy individuals (Faraji et al. 2021). Another study showed that migraine sufferers had unusual plasma cytokine levels, with higher interleukin 1‐beta (IL‐1β) and interleukin 6 (IL‐6), but lower interleukin 10 (IL‐10) (Balcziak and Russo 2022; Ha and Chu 2024). However, a 2021 study found no significant difference in IL‐1β levels between migraine patients and controls (Taheri et al. 2021). While gut microbiota is linked to migraines, the role of immune inflammation in this relationship remains unclear.

Mendelian randomization (MR) uses genetic variations, like SNPs, to explore the causal effects of gut microbiota and immune cells on migraines, reducing confounding and reverse causality. Prior MR studies have established a connection between the gut microbiome and migraine, yet they have not considered potential mediators, and the available microbiome datasets have been limited in size. This study, for the first time, employs mediated Mendelian randomization to investigate the causal relationships among bacterial taxa, immune cells, and migraine, as well as to examine the mediating role of immune cells in the impact of intestinal microbiota on migraine. The findings offer novel scientific insights into the pathogenesis of migraine and hold promise for enhancing the prediction and treatment of this condition.

## 2 Materials and Methods

### 2.1 Data Sources for Exposure and Outcome

#### 2.1.1 Genome‐Wide Association Studies (GWAS) Data Source of Gut Microbiome

Single‐nucleotide polymorphisms (SNPs) associated with gut microbiota abundance, identified at a significance threshold of p<1e‐5, were selected as genetic instrumental variables (IVs) from the extensive GWAS data conducted by the MiBioGen consortium and Netherlands Microbiome Project (DMP) (Lopera‐Maya et al. 2022). DMP were sourced from 7,738 individuals of European ancestry, while the MiBioGen consortium coordinated 16S rRNA gene sequencing profiles and genotyping data (Kurilshikov et al. 2021). A total of 211 taxa were identified, spanning from the phylum to genus levels, which included 9 phyla, 16 classes, 20 orders, 35 families, and 131 genera (Lopera‐Maya et al. 2022).

#### 2.1.2 Immunity‐Wide GWAS Data Sources

Genome‐wide association study (GWAS) summary statistics for each immune trait, determined at a significance threshold of p<5e‐8, were publicly accessible via the GWAS Catalog, with accession numbers ranging from GCST90001391 to GCST90002121 (Orrù et al. 2020). The study evaluated a total of 731 immunophenotypes in a cohort of 3,757 Sardinians, comprising 118 absolute cell (AC) counts, 389 median fluorescence intensities (MFI) indicative of surface antigen levels, 32 morphological parameters (MP), and 192 relative cell (RC) counts. An estimation was conducted on approximately 22 million SNPs genotyped using a high‐density array, employing a reference panel derived from the Sardinian sequence (Sidore et al. 2015). Subsequent correlation analyses were performed after adjusting for covariates including gender, age, and age squared.

#### 2.1.3 Genome‐Wide Association Study Data Sources for Migraine, Migraine With Aura, and Migraine Without Aura

Migraine genetic association data were sourced from the Finnish Gene6 (R9) dataset, encompassing 18,477 individuals diagnosed with migraine and 187,837 control subjects (Kurki et al. 2023). Migraine diagnoses were established in accordance with the International Classification of Diseases, utilizing codes 346 for both the Eighth and Ninth Editions (ICD‐9) and code G43 for the Tenth Edition (ICD‐10). The median age at onset for the first migraine episode was 32.88 years. Summary statistics indicated that there were 6,730 cases of MO and 287,837 controls, while MA was represented by 7,917 cases and 287,837 controls.

### 2.2 Selection of Instrumental Variables (IVs)

Mendelian randomization necessitates the fulfillment of three fundamental assumptions: (1) the genetic variants employed as instrumental variables must exhibit an association with the exposure, known as the relevance assumption; (2) the genetic variants must remain independent of any confounding variables, referred to as the independence assumption; and (3) the genetic variants should influence the outcome exclusively through the exposure, without any alternative pathways, which is termed the exclusion restriction assumption (Emdin et al. 2017). The IVs (LD r2< 0.01 and clumping distance = 10000 kb based on the European‐based 1000 Genome Projects reference panel (Hanchard and Choudhury 2022)) were selected at different significance thresholds from GWAS results of exposure. Subsequently, to mitigate weak instrument bias, SNPs with an F‐statistic value (Bowden et al. 2019) of less than 10 in the exposure GWAS were excluded. SNPs selected for MR analysis were shown in Table S1 and S2.

### 2.3 MR Analysis

**FIGURE 1:**
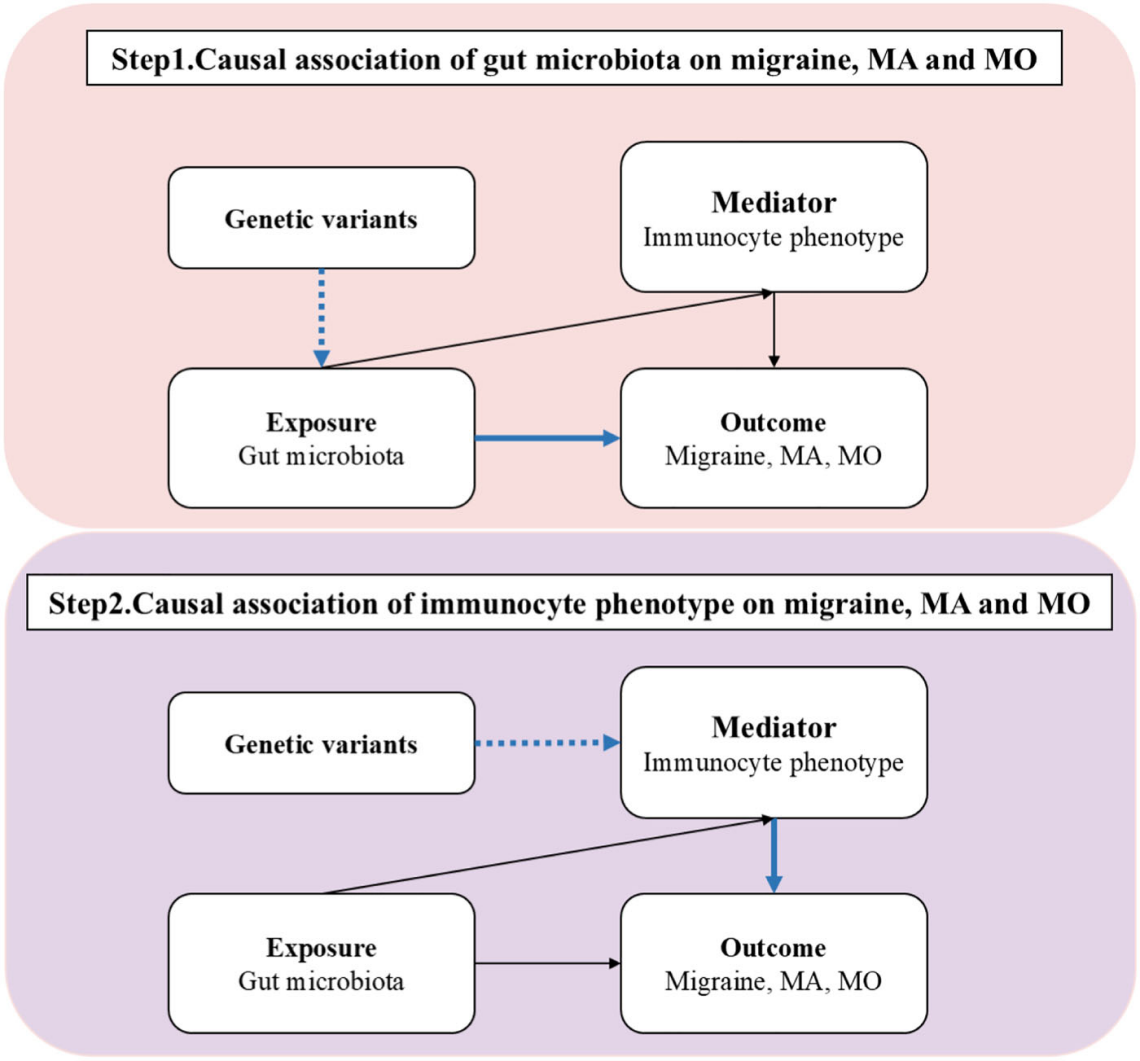
Study design overview. MA, migraine with aura; MO, migraine without aura.

In our study, we performed two‐sample Mendelian Randomization analysis to systematically assess the causal effects of gut microbiota and immunophenotypes on migraine, including its subtypes MA and MO, by using the ‘Mendelian Randomization’ package (version 0.4.3). The primary analysis was conducted using the inverse variance weighting method (Burgess et al. 2015), supported by additional analyses with MR Egger (Bowden et al. 2015), weighted median (Bowden et al. 2016), simple mode, and weighted mode methods (Hartwig et al. 2017). Furthermore, after excluding positive results containing fewer than four SNPs, we employed the MR‐PRESSO Global test (Verbanck et al. 2018) and the intercept term from the MR‐Egger method (Bowden et al. 2015) to evaluate horizontal pleiotropy. The Cochrane Q test (Burgess et al. 2013) was utilized to assess heterogeneity among the instrumental variables, with a p‐value exceeding 0.05 indicating the absence of significant heterogeneity. The MR Steiger test (Hemani et al. 2017) was employed to assess the directionality of the relationship between the exposure and the outcome. Furthermore, we used two‐step Mendelian randomization to estimate whether immunophenotypes mediate any effect of gut microbiota on migraine and its subtype risk. The study design was presented in Figure 1.

### 2.4 Animals

Male C57BL/6 mice (6 weeks) were obtained from Huaxia Kaiqi Biotechnology Co., Ltd. All mice were housed under a 12/12 light cycle with ad libitum access to food/water. All procedures were approved by the Ethics Committee of Southern Medical University. The migraine model was induced by intraperitoneal injection of nitroglycerin (NTG, 10 mg/kg diluted in saline) every other day for five times, while control mice received equivalent volumes of saline(Figure 6A) (Pradhan et al. 2014).

### 2.5 Mechanical Threshold Test

The thresholds for mechanical were measured 2 h before each injection. Mice were placed in a glass box and allowed to acclimate for 30 min prior to testing. A series of calibrated von Frey filaments (bending force range: 0.02–4.0 g) were applied perpendicularly to the plantar surface of the hind paws. The withdrawal threshold was assessed by recording the paw's immediate withdrawal response to applied pressure at the tip, with the final value calculated as the average of three consecutive trials.

### 2.6 qPCR

Feces DNA was extracted with a DNA isolation kit (Solarbio, Beijing, China). Reactions were carried out on a LightCycler 480 II Detection System (Roche) using SYBR Premix Taq Pro Universal (Vazyme, Q712‐02). Gene expression values were standardized against 16S as the endogenous control.

The primer sequences were as follows: 16S, 5′‐ACTCCTACGGGAGGCAGCAGT‐3′ and 5′‐ATTACCGCGGCTGCTGGC‐3′; *Prevotella histicola*, 5′‐ CACGTGTGATTGTTTGCAGGT‐3′, 5′‐TCCAGCCTACGCTCCCTTTA‐3′.

## 3 Results

### 3.1 Causal Effect of Gut Microbiome on Migraine, Migraine, MA, and MO

**FIGURE 2:**
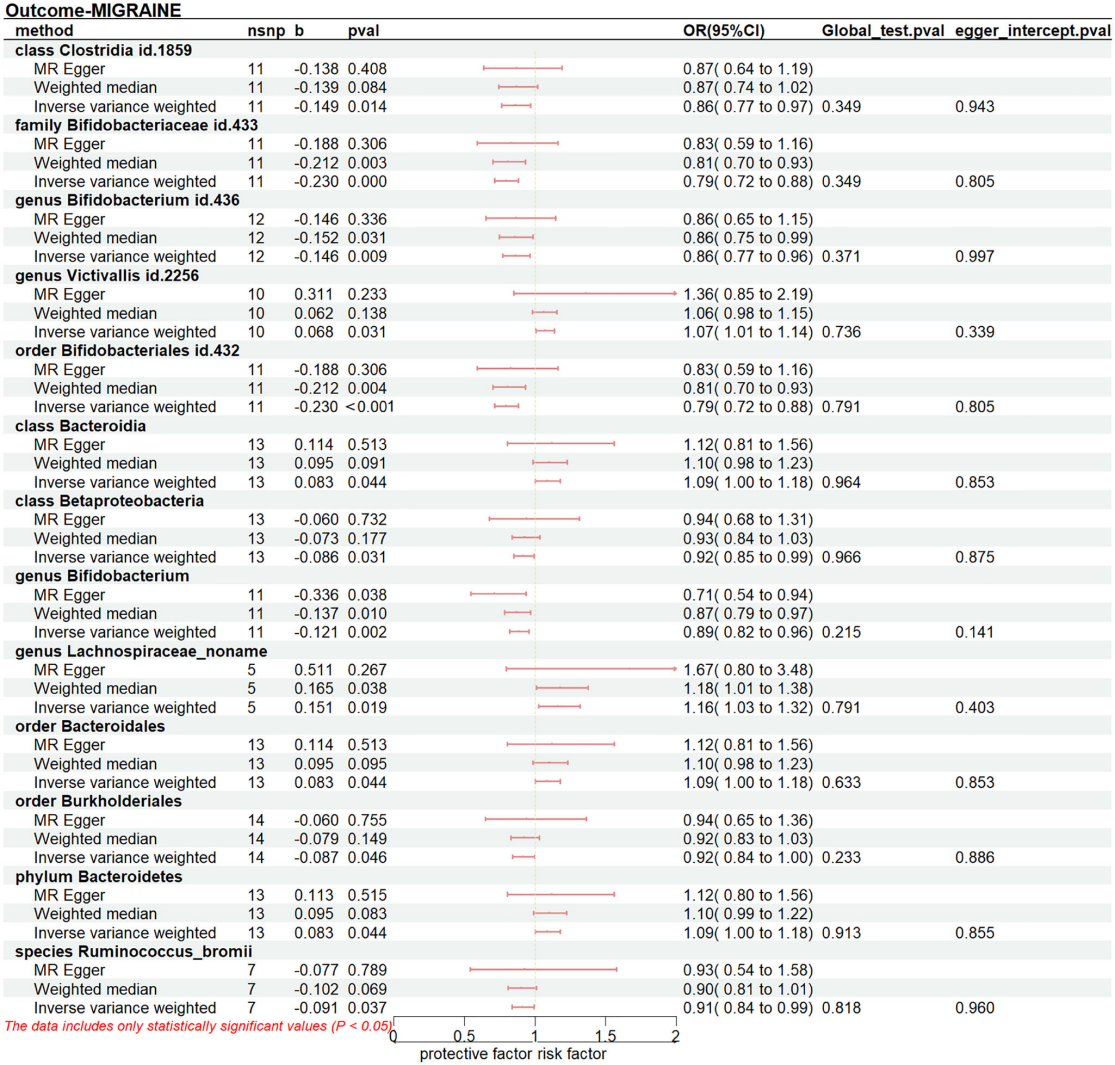
Forest plot presents the causal relationship between gut microbiota and migraine. Nsnp, number of single‐nucleotide polymorphisms; b, beta; OR, odds ratio; CI, confidence intervals.

**FIGURE 3:**
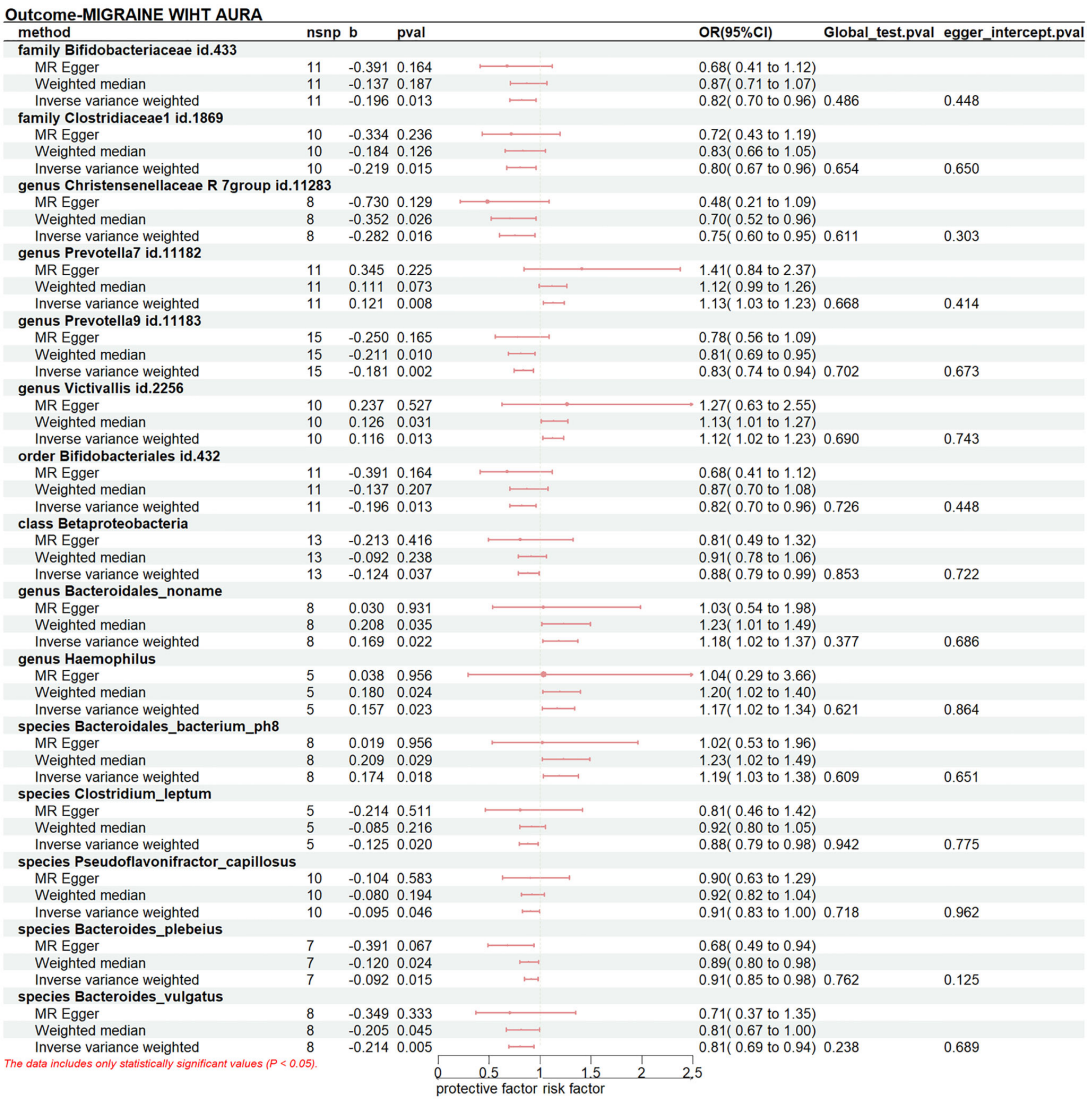
Forest plot presents the causal relationship between gut microbiota and migraine with aura. Nsnp, number of single‐nucleotide polymorphisms; b, beta; OR, odds ratio; CI, confidence intervals.

**FIGURE 4:**
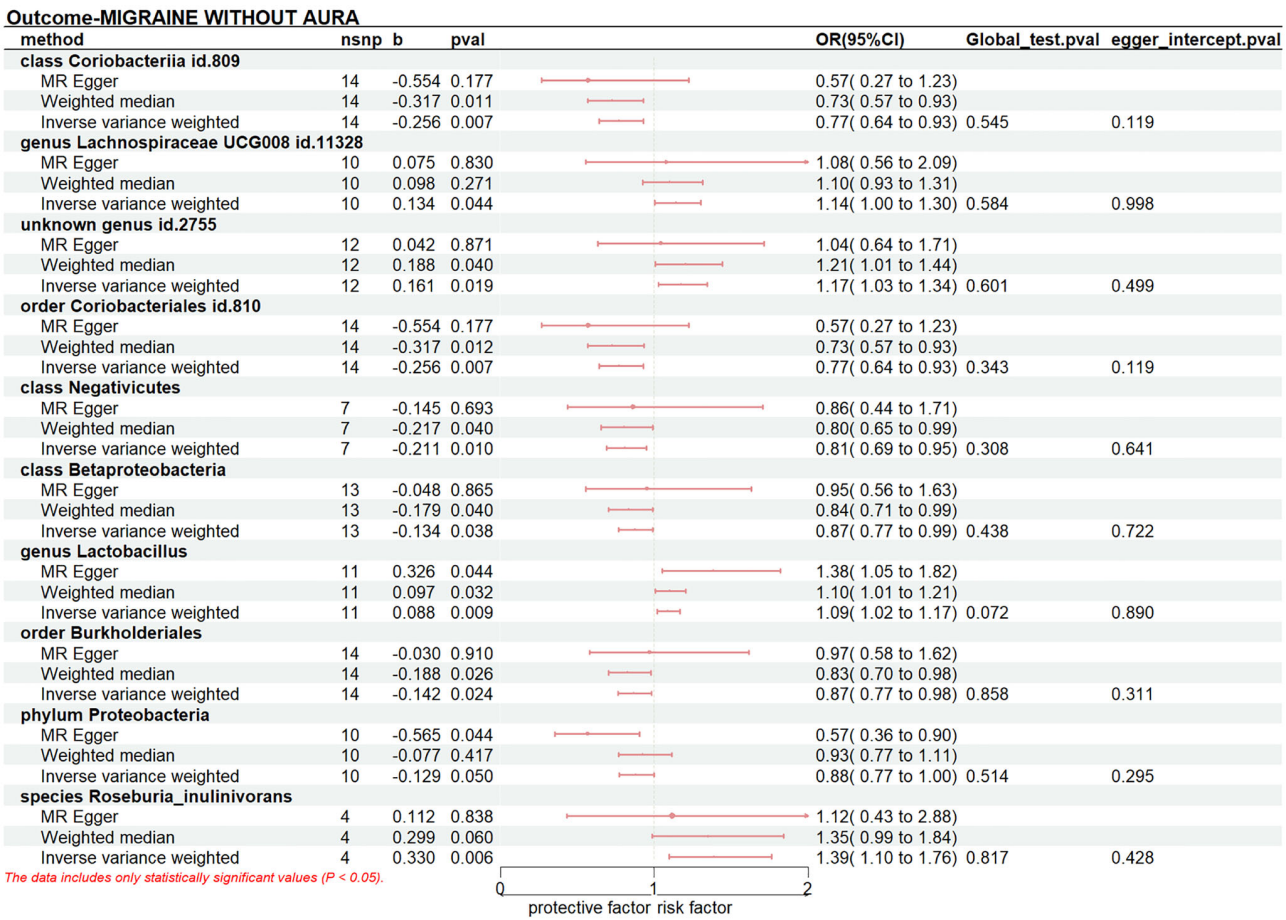
Forest plot presents a causal relationship between gut microbiota and migraine without aura. Nsnp, number of single‐nucleotide polymorphisms; b, beta; OR, odds ratio; CI, confidence intervals.

Gut microbiome taxa's significant results on migraine, MA, and MO were shown in Figures 2, 3, 4. The results demonstrated that genus *Victivallis* id.2256(P_IVW_:0.031, OR_IVW_:1.070, 95% CI:1.006‐1.138), class *Bacteroidia* (P_IVW_:0.044, OR_IVW_:1.087, 95% CI:1.002‐1.178), genus *Lachnospiraceae noname* (P_IVW_: 0.020, OR_IVW_: 1.163, 95% CI:1.023‐1.319), order *Bacteroidales* (P_IVW_: 0.044, OR_IVW_: 1.087, 95% CI:1.002‐1.178), phylum *Bacteroidetes* (P_IVW_: 0.044, OR_IVW_: 1.086, 95% CI:1.002‐1.178) were associated to a higher risk of migraine, while class *Clostridia id.1859*(P_IVW_: 0.014, OR_IVW_: 0.861, 95% CI:0.765 ‐0.970), family *Bifidobacteriaceae id.43*3(P_IVW_:1.51×10^−5^, OR_IVW_: 0.861, 95% CI:0.765 ‐0.970), genus *Bifidobacterium id.436*(P_IVW_:0.009, OR_IVW_: 0.864, 95% CI:0.774 ‐0.965), order *Bifidobacteriales id.432* (P_IVW_:1.51×10^−5^, OR_IVW_: 0.861, 95% CI:0.765 ‐0.970), class *Betaproteobacteria* (P_IVW_:0.030, OR_IVW_: 0.917, 95% CI:0.848 ‐0.992), genus *Bifidobacterium* (P_IVW_:0.002, OR_IVW_: 0.886, 95% CI:0.821 ‐0.957), order *Burkholderiales* (P_IVW_:0.046, OR_IVW_: 0.916, 95% CI:0.841 ‐0.999), species Ruminococcus_bromii (P_IVW_:0.037, OR_IVW_: 0.913, 95% CI:0.838 ‐0.995) decreased the risk of migraine.(Figure 2, Table S3)

The increased level of genus Prevotella7 id.11182 (P_IVW_: 0.008, OR_IVW_: 1.129, 95% CI:1.032‐1.235), genus Victivallis id.2256 (P_IVW_: 0.013, OR_IVW_: 1.123, 95% CI: 1.025‐1.231), genus Bacteroidales_noname (P_IVW_: 0.022, OR_IVW_: 1.184, 95% CI: 1.024‐1.367), genus Haemophilus (P_IVW_: 0.023, OR_IVW_: 1.170, 95% CI: 1.022‐1.340), species *Bacteroidales_bacterium_ph8* (P_IVW_:0.018, OR_IVW_:1.190, 95% CI: 1.230‐1.375) increased the risk of MA. Conversely, we detected protective effects of ten taxa on MA: family *Bifidobacteriaceae id.433* (P_IVW_:0.013, OR_IVW_: 0.821, 95% CI:0.704 ‐0.960), family *Clostridiaceae1 id.1869*(P_IVW_:0.015, OR_IVW_: 0.803, 95% CI:0.67 ‐0.958), genus *Christensenellaceae id.11283* (P_IVW_:0.016, OR_IVW_: 0.754, 95% CI:0.599‐0.950), *genus Prevotella9 id.11183* (P_IVW_:0.002, OR_IVW_: 0.834, 95% CI:0.744 ‐0.936), order *Bifidobacteriales id.432* (P_IVW_:0.013, OR_IVW_: 0.821, 95% CI:0.704 ‐0.960), class *Betaproteobacteria*(P_IVW_:0.037, OR_IVW_: 0.884, 95% CI:0.787 ‐0.993), species *Clostridium_leptum*(P_IVW_:0.020, OR_IVW_: 0.882, 95% CI:0.794 ‐0.980), species *Pseudoflavonifractor_capillosus*(P_IVW_:0.020, OR_IVW_: 0.882, 95% CI:0.794 ‐0.980), species *Bacteroides_plebeius*(P_IVW_:0.015, OR_IVW_: 0.912, 95% CI:0.848 ‐0.982), species *Bacteroides_vulgatus*(P_IVW_:0.005, OR_IVW_: 0.807, 95% CI:0.695 ‐0.938).(Figure 3, Table S4)

The results indicated a causal effect between a higher liability of MO and an increased level of genus *Lachnospiraceae UCG008 id.11328* (P_IVW_: 0.044, OR_IVW_: 1.143, 95% CI: 1.003‐1.304), *unknown genus id.2755* (P_IVW_: 0.019, OR_IVW_: 1.174, 95% CI: 1.027‐1.343), genus *Lactobacillus* (P_IVW_: 0.009, OR_IVW_: 1.092, 95% CI: 1.022‐1.166), and species *Roseburia_inulinivorans* (P_IVW_: 0.006, OR_IVW_: 1.391, 95% CI: 1.097‐1.763). However, class *Coriobacteriia id.809* (P_IVW_: 0.005, OR_IVW_: 0.807, 95% CI: 0.695‐0.938), order *Coriobacteriales id.810* (P_IVW_: 0.007, OR_IVW_: 0.774, 95% CI: 0.643‐0.932), class *Negativicutes* (P_IVW_: 0.010, OR_IVW_: 0.810, 95% CI: 0.690‐0.950), class *Betaproteobacteria* (P_IVW_: 0.038, OR_IVW_: 0.875, 95% CI: 0.771‐0.993), order *Burkholderiales* (P_IVW_: 0.024, OR_IVW_: 0.868, 95% CI: 0.768‐0.981), phylum *Proteobacteria* (P_IVW_: 0.050, OR_IVW_: 0.879, 95% CI: 0.773‐1.000) have protective causal relationships with the risk of MO.(Figure 4, Table S5)

### 3.2 Causal Effect of Immune Traits on Migraine, MA, and MO

For migraine, it was observed that CD27 on memory B cells (P_IVW_: 0.023, OR_IVW_: 1.043, 95% CI: 1.005‐1.083) and CD27 on unswitched memory B cell (P_IVW_: 0.023, OR_IVW_: 1.043, 95% CI: 1.005‐1.083) CD40 on CD14‐ CD16+ monocyte (P_IVW_: 0.023, OR_IVW_: 1.043, 95% CI: 1.005‐1.083) were significantly associated with an increased risk. Instead, CD40 on CD14‐CD16+ monocyte (P_IVW_: 0.034, OR_IVW_: 0.965, 95% CI: 0.933‐0.997) and CD8 on Effector Memory CD8+ T cell (P_IVW_: 7.24×10^−4^, OR_IVW_: 0.921, 95% CI: 0.878‐0.966) were found to reduce the risk of migraine.

A higher genetic prediction of HLA DR on CD14+ CD16‐ monocyte (P_IVW_: 0.028, OR_IVW_: 1.055, 95% CI: 1.006‐1.106), HLA DR on CD14+ monocyte (P_IVW_: 0.028, OR_IVW_: 1.057, 95% CI: 1.006‐1.110) and HLA DR on monocyte (P_IVW_: 0.001, OR_IVW_: 1.076, 95% CI: 1.029‐1.125) increase the risk of MA. In contrast, the level of CD8 on Effector Memory CD8+ T cell (P_IVW_: 0.001, OR_IVW_: 0.885, 95% CI: 0.821 ‐0.954) was associated with a lower risk of MA.

**FIGURE 5:**
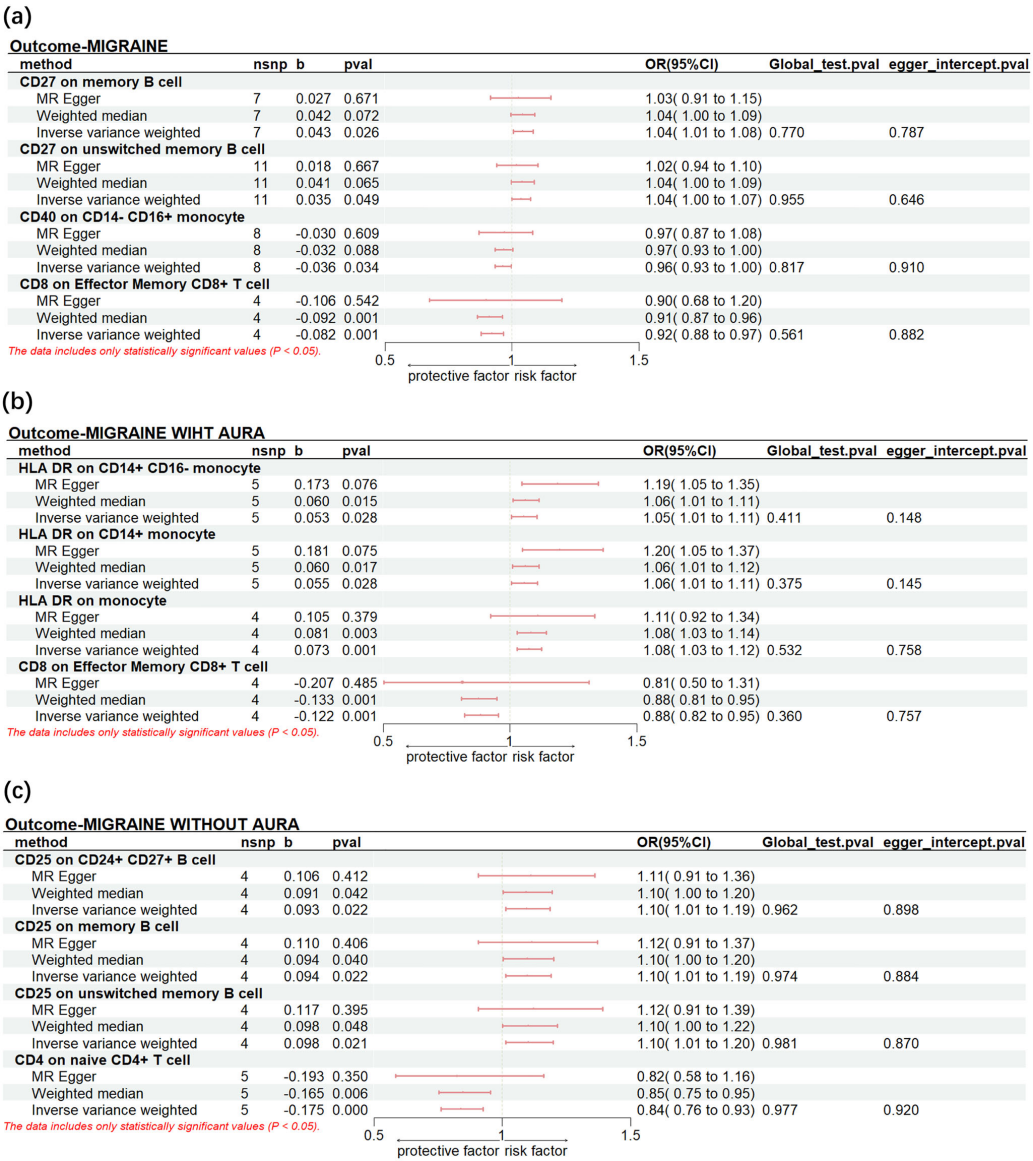
The forest plot presents the causal relationship between immunocyte phenotype and migraine (a), MA (b), and MO (c). Nsnp, number of single‐nucleotide polymorphisms; b, beta; OR, odds ratio; CI, confidence intervals.

We observed genetic predisposition to CD25 on CD24+ CD27+ B cell (P_IVW_: 0.022, OR_IVW_: 1.097, 95% CI: 1.013‐1.187), CD25 on memory B cell (P_IVW_: 0.022, OR_IVW_: 1.099, 95% CI: 1.014‐1.191) and CD25 on unswitched memory B cell (P_IVW_: 0.021, OR_IVW_: 1.103, 95% CI: 1.015‐1.199) were associated with a higher risk for MO. However, CD4 on naive CD4+ T cell (P_IVW_: 4.512×10^−4^, OR_IVW_: 0.839, 95% CI: 0.761 ‐0.926) decreased risk of MO significantly. The significant result was shown in Figure 5

### 3.3 Mediation Analyses of Potential Immune Traits

In the two‐step Mendelian Randomization, we found *Prevotella* exerted protective effects against MA by downregulating HLA‐DR on monocyteS. HLA‐DR on monocyteS mediated 7.74% of the causal pathway from genus *Prevotella9 id.11183* (PIVW: 0.002, ORIVW: 0.834, 95% CI: 0.744‐0.936) to MA.

### 3.4 Sensitivity Analyses

The MR‐Egger regression intercept approach showed that genetic pleiotropy did not bias the outcomes, and MR‐PRESSO analysis proved there was no horizontal pleiotropy in the MR study (P  >  0.05).

### 3.5 Experimental Validation

**FIGURE 6:**
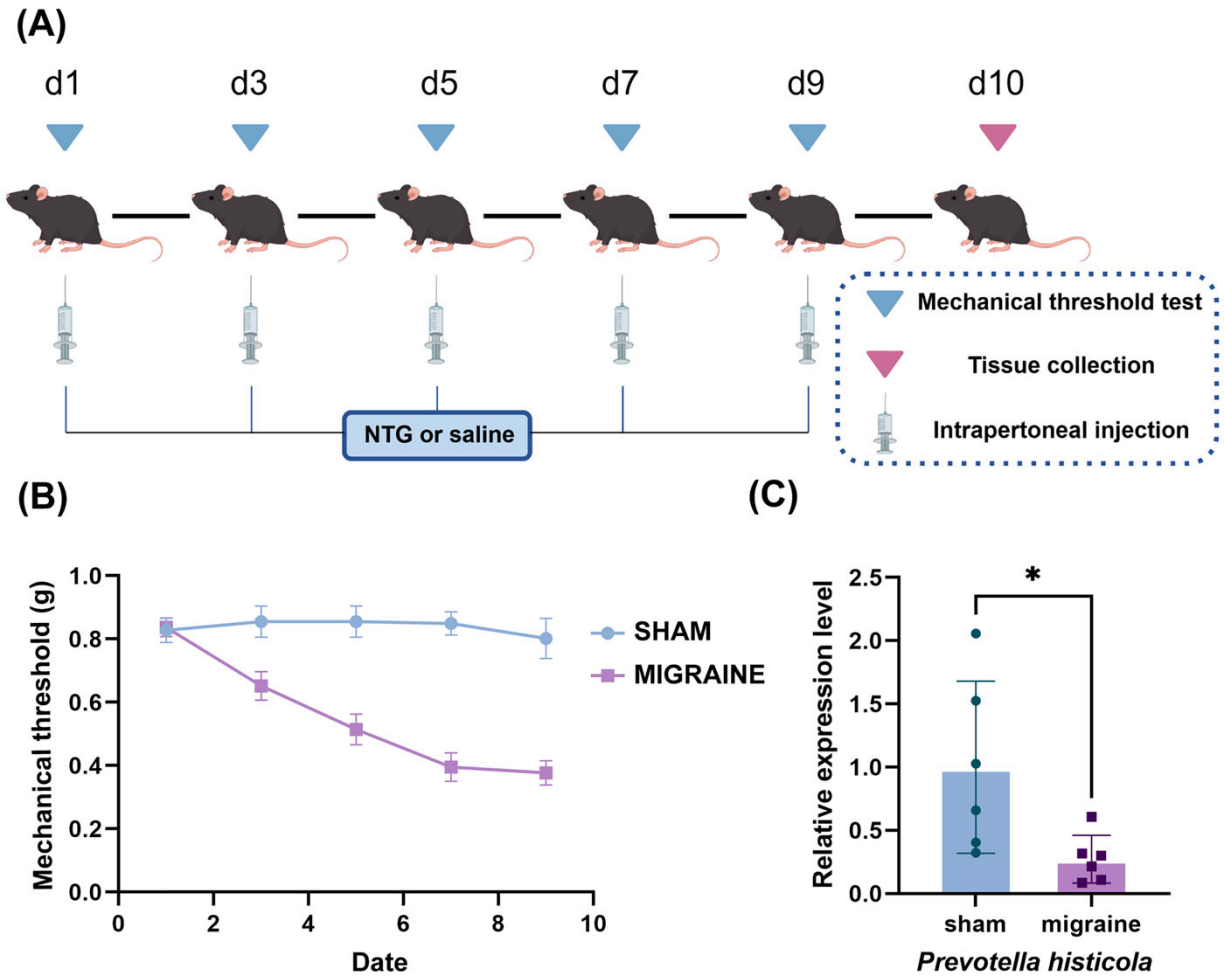
(A) Time schedule of (nitroglycerin) NTG administration in mice. (B) Development of mechanical withdrawal thresholds of paws following NTG or saline injection. (C) The abundance of Prevotella histicola was significantly increased in the migraine group compared with the sham group. Statistical analysis was performed using a t‐test, n  =  6, *p <  0.05 compared to the sham group.

Clinically, cutaneous allodynia is a common manifestation in migraineurs. We observed a gradual decrease in mechanical withdrawal thresholds of paws following NTG injection (Figure 6B). To assess differences in *Prevotella* abundance between migraine‐model and control mice, fecal qPCR analysis was performed (Figure 6C). Migraine‐model mice exhibited a significant reduction in fecal *Prevotella* levels compared to controls, aligning with prior Mendelian randomization data.

## 4 Discussion and Conclusion

In this study, we employ a two‐step MR approach to elucidate (1) the causal relationship between gut microbiota and migraine, including its subtypes; (2) the causal relationship between immunocyte phenotypes and gut microbiota; and (3) the mediation effects of these immunocyte phenotypes. Initially, we identified a potential association between gut microbiota and migraine, as well as its subtypes, MA and MO. Our analysis revealed that specific gut bacterial strains, numbering 13, 15, and 12, respectively, may contribute to the pathogenesis of migraine. The protective role of *Bifidobacterium* in migraine was statistically significant. Our findings indicate that Betaproteobacteria may reduce the risk of migraines, MA, and MO, while an increased abundance of certain bacteria, such as *Bifidobacterium* and *Clostridiaceae*, might confer protection against migraine and MA. Similarly, *Burkholderiales* appears to exert a protective effect against both migraine and MO. Conversely, *Bacteroidetes* and *Fusobacterium* are identified as risk factors for migraine, MA, and MO. Furthermore, our study underscores the presence of Prevotella, which merits further investigation. Further mediation analysis using Mendelian randomization suggests that Prevotella may influence MA through alterations in HLA‐DR expression on monocytes. Finally, we validated the reduced abundance of *Prevotella histicola* in fecal samples of migraine model mice using qPCR assays, and the results were consistent with the Mendelian randomization analysis.

Migraine, a neurological disorder characterized by moderate to severe headache as its core symptom, has garnered increasing attention in recent years (Raggi et al. 2024). Emerging evidence from multiple studies suggests a potential association between migraine and gastrointestinal disorders (Arzani et al. 2020). Gut dysbiosis may contribute to increased migraine risk through mechanisms including elevated release of pro‐inflammatory factors, dysregulated neuropeptides (e.g., glutamate), and heightened calcitonin gene‐related peptide (CGRP) signaling (Vuralli et al. 2024; Farzi et al. 2018; Ramachandran 2018). Furthermore, clinical observations indicate that therapeutic interventions targeting gut microbiota modulation—such as dietary modifications and probiotic supplementation—may confer beneficial effects on migraine pathophysiology (Jibril et al. 2022, Martami et al. 2019). Previous research has demonstrated a significant association between gut microbiota composition and the incidence of migraines (Tang et al. 2020; Chen et al. 2020; Wen et al. 2019; Yong et al. 2023). Observational studies have revealed that, in comparison to elderly women suffering from migraines, healthy control groups exhibit a higher prevalence of beneficial microbial species, including *Faecalibacterium prausnitzii*, *Bifidobacterium adolescentis*, and *Methanobrevibacter smithii* (Chen et al. 2020). Additionally, fecal 16S rRNA sequencing has shown a marked difference in the abundance of *Bifidobacterium* between normal and migraine‐afflicted mice (Wang et al. 2024). *Bifidobacteria*, classified under the *phylum Actinobacteria* and the class *Bifidobacteria*, are recognized as beneficial microbes (Ventura et al. 2012; Fukuda et al. 2011). One study has demonstrated that exopolysaccharides produced by *Bifidobacterium* are linked to a reduction in the expression of inflammatory cytokines, such as TNF‐α, IL‐6, and IL‐12a, in murine bone marrow‐derived macrophages (Hickey et al. 2021). Importantly, recent investigations have reported elevated levels of inflammatory cytokines, including TNF‐α and IL‐6, during migraine episodes (Ha and Chu 2024). Consequently, *Bifidobacterium* may confer a protective effect against migraines by modulating the release of these immune factors.

Our findings suggest that HLA‐DR expression on monocytes may serve as an intermediary in mediating the causal relationship between *Prevotella9 id.11183* and MA. *Prevotella*, a genus of Gram‐negative anaerobic bacteria prevalent in the oral cavity, intestines, and vagina, plays a significant role in metabolic pathways (Tett et al. 2021). Previous studies have indicated that the relative abundance of *Prevotella* decreases in rats treated with Tianma for migraine, a phenomenon hypothesized to be closely linked to amino acid metabolism (Wen et al. 2019). Additionally, it has been reported that *Prevotella copri* produces short‐chain fatty acids (SCFAs), which may protect the mucosal barrier and mitigate inflammation (Bedarf et al. 2017). Furthermore, recent research has demonstrated that transplantation of *Prevotella copri* can reshape the gut microbiota, alleviate oxidative stress, and reduce neurological impairment following traumatic brain injury (Gu et al. 2024). Collectively, these studies underscore the potential relationship between *Prevotella* abundance, immune mechanisms, and the mitigation of inflammatory responses. Notably, reduced expression of HLA‐DR on monocytes is recognized as a marker of altered immune status in patients experiencing systemic inflammatory responses (Kim et al. 2010). Consequently, we hypothesize that *Prevotella* may influence the reduction of MA incidence in the population by modulating SCFA metabolism, oxidative stress, and other related factors, thereby diminishing HLA‐DR expression on monocytes. To date, limited research has investigated the mechanisms underlying the impact of Prevotella on MA, and further basic research is required to elucidate these specific mechanisms.

The strengths of this study are as follows: (1) It introduces a novel perspective by employing two‐step MR for the first time to investigate the influence of gut microbiota on migraine through immune phenotypes, thereby providing new insights into the mechanisms underlying migraine occurrence. (2) The study utilizes the latest large‐scale GWAS data, incorporating an extensive range of gut microbiota data to enhance the robustness of the findings. (3) A two‐sample Mendelian randomization design was adopted, which, in comparison to observational studies, significantly reduces bias arising from confounding factors and reverse causality.

Our Mendelian Randomization analysis is subject to several limitations. Firstly, despite employing multiple sensitivity analyses to mitigate bias, it is improbable that bias has been entirely eradicated. Secondly, it is important to acknowledge that the GWAS statistics utilized in our study are derived exclusively from individuals of European descent, thereby limiting the generalizability of our findings to other populations. Future research should incorporate data from more diverse populations to enhance the applicability of the results. Thirdly, the lack of access to individual‐level data precludes the possibility of conducting stratified analyses, which would enable the derivation of more precise outcomes.

## Author Contributions

**Yupei Lai**: conceptualization, data curation, investigation, writing ‐ original draft, writing ‐ review and editing, formal analysis, methodology. **Yike Liu**: software, data curation. **Jiahao Chen**: methodology, resources. **Yu Cao**: conceptualization, investigation. **Xiangsheng Zhang**: investigation, data curation. **Lu Li**: software, methodology. **Pengyu Zhou**: visualization, validation. **Peng Sun**: project administration, writing ‐ review and editing. **Jun Zhou**: writing ‐ review and editing, conceptualization, methodology, funding acquisition, project administration, supervision.

## Conflicts of Interest

The authors declare no conflicts of interest.

## Peer Review

The peer review history for this article is available at https://publons.com/publon/10.1002/brb3.70693

## Supporting information




**Supporting Material**: brb370693‐sup‐0001‐SupMat.xlsx

## Data Availability

The datasets analyzed in this study are publicly available summary statistics.
